# Mutant LRRK2 in lymphocytes regulates neurodegeneration via IL-6 in an inflammatory model of Parkinson’s disease

**DOI:** 10.1038/s41531-022-00289-9

**Published:** 2022-03-15

**Authors:** Elena Kozina, Matthew Byrne, Richard Jay Smeyne

**Affiliations:** Department of Neurosciences, Jefferson Hospital for Neuroscience, Thomas Jefferson University, 900 Walnut St, Philadelphia, PA 19107 USA

**Keywords:** Parkinson's disease, Neuroimmunology

## Abstract

Mutations in a number of genes contribute to development of Parkinson’s disease (PD), including several within the LRRK2 gene. However, little is known about the signals that underlie LRRK2-mediated neuronal loss. One clue resides in the finding that the neurodegenerative cascades emanate from signals arising from the peripheral immune system. Here, using two chimeric mouse models, we demonstrate that: 1) the replacement of mutant LRRK2 with *wt* form of the protein in T- and B-lymphocytes diminishes LPS-mediated inflammation and rescues the SNpc DA neuron loss in the mutant LRRK2 brain; 2) the presence of G2019S or R1441G LRRK2 mutation in lymphocytes alone is sufficient for LPS-induced DA neuron loss in the genotypically *wt* brain; and 3) neutralization of peripheral IL-6 overproduction prevents the SNpc DA neuron loss in LPS-treated mutant LRRK2 mice. These results represent a major paradigm shift in our understanding of PD pathogenesis and suggest that immune dysfunction in some forms of familial PD may have primacy over the CNS as the initiating site of the disorder.

## Introduction

Parkinson’s disease (PD), the second most common neurodegenerative disorder, is a major public health predicament affecting over 6 million people^1^ with estimates that this number will rise to 12 million by 2040^2^. Pathologically within the CNS, PD is characterized by the progressive loss of dopamine (DA) neurons in the substantia nigra pars compacta (SNpc), the presence of intracellular α-synuclein-positive inclusions (Lewy bodies), and decreased DA in the striatum^3^. PD also manifests with other non-motor symptoms including olfactory impairment, depression, autonomic dysfunction, sleep disturbances, and gut dysbiosis^4^. Symptomatic treatment for the motor symptoms of PD primarily involves DA-replacement therapy, but no currently available treatment has been demonstrated to stop or slow down the progression of this disease. Although PD has been historically viewed as a brain-specific disease, it is clear that it also affects other systems and organs. Recent evidence from clinical and experimental studies suggests an influential, yet largely underappreciated, force in PD pathogenesis, that of immune signals originating outside the brain. Indeed, one of the most common features of both familial and sporadic PD is dysregulated peripheral immunity^5–10^. Numerous human studies over the past years have reported alterations in cytokines in PD patients biofluids (both, serum and CSF)^11^, impaired monocytes phagocytic activity^12^, a distinct transcriptomic profile of peripheral monocytes^13^, an unbalance in Th cells toward the pro-inflammatory phenotypes^14^, decrease in Treg ability to suppress the activity of Teff cells^15–17^ and the presence of α-synuclein reactive T-cells in the blood of newly diagnosed PD patients^18^. In addition, GWAS have recently implicated several SNPs in HLA-DR regions which are associated with late-onset idiopathic PD further indicating a role for the immune system in PD susceptibility^19^. There is also growing awareness within the scientific community of the strong connection between intestinal inflammation and the changes in the composition of intestinal bacterial populations related to PD pathogenesis^20–22^. Recent studies also demonstrated increased levels of endotoxin in PD patients’ blood, especially those with a higher risk for dementia, as well as greater gastrointestinal permeability with lower levels of LPS-binding protein and increased gut staining for *E. coli* suggesting an active role for bacterial infection in the outcome of the disease^23,24^. Thus, it is now proposed that the immune component in PD occurs early and changes dynamically with the disease progression, contributing to the neuronal loss observed in patients.

While the vast majority of PD cases arise from unknown causes, about 10–15% have a clear genetic etiology^25^. At this time, over 90 loci have been associated with PD^26^, although only a small number of them, including the gene encoding Leucine Rich Repeat Kinase 2 (LRRK2), account for the majority of familial cases^27^. LRRK2 is known to express in both innate and adaptive immune cells and its expression is tightly regulated by immune stimulation^28–34^. It has also been shown that LRRK2 expression is significantly increased in inflamed intestinal tissue biopsied from patients with Crohn’s disease, indicating that LRRK2 might play a more generalized role in the inflammatory process than previously recognized^28^. A number of studies have shown that peripheral pro-inflammatory cytokine responses to challenge were altered in mice carrying the LRRK2 mutation^35–37^ as well as in asymptomatic PD patients carrying a G2019S LRRK2 mutation^38^; suggesting a role for LRRK2 in peripheral inflammation. Our group has recently shown that mice carrying two most common pathogenic LRRK2 mutations, G2019S or R1441G, develop a significantly higher LPS-induced SNpc DA cell loss. We also found that the exacerbated neuroinflammation and SNpc DA cell loss do not depend on the active participation of the brain resident microglia or infiltrating T-cells and/or monocytes, but are likely mediated by circulating inflammatory molecules that are dysregulated by mutant LRRK2^37^. Based on these studies, we now propose that the neurodegenerative cascades in PD emanate from signals arising from the peripheral immune system and that LRRK2-PD is primarily an immune disorder, with secondary effects manifested in the CNS. Since the majority of PD studies focus on the events within the CNS and the contribution of peripheral immune compartments is largely ignored, in this study we have asked a fundamental question: is altered peripheral immune response alone sufficient to initiate neuronal loss in LRRK2-PD?

To test this hypothesis, we generated chimeric mice with a *wt* adaptive immune system and a LRRK2 mutant brain (and vice versa) and then examined whether this in vivo “switching” impacts both peripheral and CNS inflammatory response and SNpc DA cell loss following exposure to LPS. Herein, we report that the replacement of mutant LRRK2 with *wt* LRRK2 in peripheral T- and B-cells diminishes the peripheral inflammatory response to LPS and rescues SNpc DA neuron loss that is observed in fully mutant LRRK2 mice. Additionally, “reversed” chimera mice carrying normal *wt* LRRK2 in the brain but mutant LRRK2 (R1441G or G2019S) in the adaptive immune cells do manifest the LPS-induced SNpc DA neuron loss identical to LPS-treated fully mutant LRRK2 mice. Furthermore, we identified peripheral IL-6 as a key component of inflammation-mediated neuronal loss since neutralization of LPS-induced IL-6 overproduction in R1441G LRRK2 mutants completely abolishes the SNpc DA cell loss. Based on these data we suggest that LRRK2-induced immune dysfunction in PD is not simply a manifestation of the pathology, but primarily drives the development of the disorder, and propose the provocative hypothesis that LRRK2-PD has its etiologies in a malfunction of the adaptive immune response.

## Results

### Generation of BM chimeras and characterization of immune phenotype

Given that irradiation severely damages both innate and adaptive immune systems as well as the CNS^39–43^, we first decided to verify whether efficient donor cells engraftment can be achieved in homozygous immunodeficient Rag1^−/−^ recipients without a pre-conditioning regimen (Supplementary Fig. 1a). Rag1^−/−^ mice lack mature T- and B-lymphocytes as a result of the deficit in V(D)J recombination^44^. In contrast to SCID mice, which develop some functional lymphocytes with age, the phenotype of Rag1^−/−^ mice is described as a “non-leaky” immunodeficiency which makes this strain an ideal candidate for immune repopulation via bone marrow transplantation (BMT). Furthermore, Rag1^−/−^ mice have previously been examined for evidence of any inherent neuropathology, and no histopathological (including the loss of nigral DA neurons or microglia activation) or behavioral abnormalities related to a PD phenotype were observed^45,46^.

Flow cytometry analysis of peripheral mononuclear cells and splenocytes isolated from Rag1 chimeras 8 weeks post-transplantation with GFP BM cells revealed that reconstitution efficacy (% of GFP-positive cells/total live cells) was about 80% in both PBMCs (Supplementary Fig. 1b, c) and spleen (data not shown). Importantly, only CD19+, CD4+, and CD8 + cells were GFP-positive, while CD11b + cells (monocytes) were GFP-negative suggesting that the host innate immune system remained intact after BMT (Supplementary Fig. 1d). Since Rag1^−/−^ mice fail to generate antibodies due to impaired B-cell development^44^, we next assessed whether antibody production was restored in BM-transplanted mice. We found that 8 weeks post-BMT, antibody production in chimeras completely returned to the WT level (Supplementary Fig. 2a). To ensure that cytokines, which are over-produced in Rag1^−/−^ due to the lack of T-cell mediated inhibition of innate immunity^47^, also returned to the normal level in BM-transplanted chimeras, we next measured serum cytokines and chemokines in naïve and LPS-treated Rag1 chimeric mice. We showed that BMT normalized both baseline and stimuli-induced cytokine response in Rag1 chimeras to the WT level (Supplementary Fig. 2b, c).

### Generation of double mutant chimeras carrying wt adaptive immune system

**Fig. 1:**
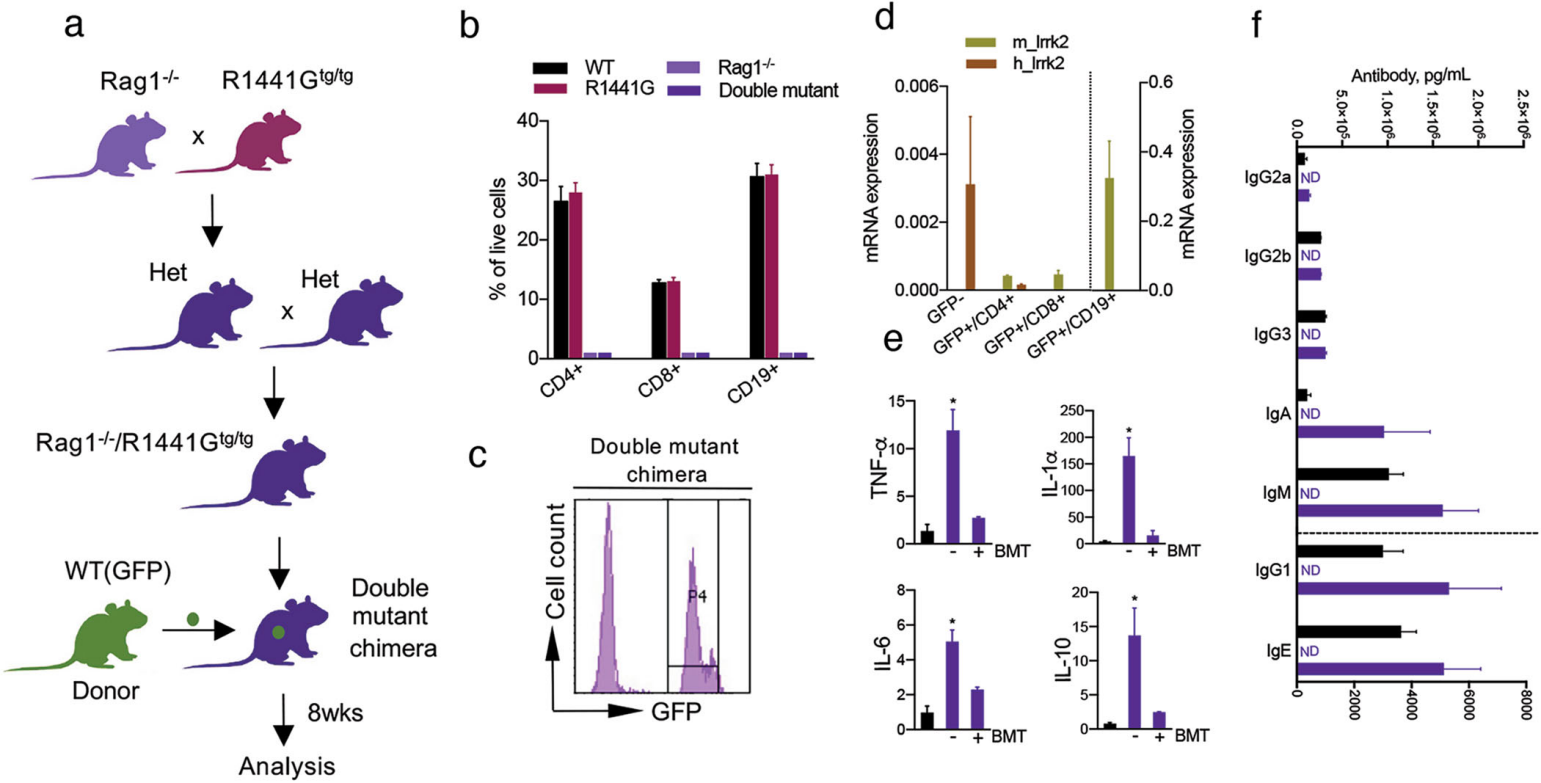
Breeding paradigm and immune phenotype characterization of double mutant chimeras. **a** Breeding diagram and BMT strategy used to generate double mutant chimeras, (WT)GFP ->Rag1/R1441G. **b** Flow cytometry confirmed that similar to Rag1^−/−^ mice, non-transplanted double mutant mice lack both B- and T-lymphocytes regardless of R1441G transgene expression. Note that immune-competent R1441G mice used as a control exhibit normal proportions of adaptive immune cells compared to WT. Data are mean ± SEM, *n* = 20 for WT and R1441G groups; *n* = 3–4 for Rag1^−/−^ and double mutant groups. **c** Representative flow cytometry plot from blood of double mutant chimera 8 weeks after BMT demonstrating significant repopulation with GFP + BM cells. **d** qPCR analysis showed the lack of human *lrrk2* mRNA expression in T- and B-cells sorted from BM-transplanted double mutant chimera’s (WT(GFP) -> Rag1/R1441G). Cells were sorted based on GFP+/CD4 + (CD4-PerCP-CY5.5), GFP+/CD8 + (CD8-PE-CY7) and GFP+/CD19 + (CD19-APC-CY7) fluorescent intensity. Sorted GFP- cells from double mutant chimeras (innate immune cells) were used as a positive control for human *lrrk2* mRNA expression. mRNA expression was normalized to mouse β-actin. Data are mean ± SEM, *n* = 4. **e**, **f** Similar to Rag1 chimeras, serum cytokines, and antibodies are back to control R1441G level in BM-transplanted double mutant chimeras. Data are mean ± SEM, *n* = 5–10 for each group. ND not detectable.

Having shown that chimerism and functional adaptive immune response can be achieved in transplanted Rag1^−/−^ mice, we next crossed Rag1^−/−^ mice to transgenic LRRK2 mice overexpressing the human pathogenic R1441G mutation, and then intercrossed these heterozygous mice to generate double mutant mice (Fig. 1a). Genotypes of double-mutant mice were confirmed in each experiment and only homozygous Rag1^−/−^/R1441G^tg/tg^ were used in the study. To confirm that the resulting double mutants lacked T- and B-cells similar to the parental Rag1^−/−^ strain, flow cytometry was performed on peripheral mononuclear cells extracted from 3-month old mice. Analysis confirmed that double mutant animals, regardless of LRRK2 transgene expression, lacked CD4+, CD8 + T-cells as well as CD19 + B lymphocytes (Fig. 1b). It is also important to note that the number of these cells was unchanged in immune-competent non-treated R1441G mice compared to WT mice suggesting that mutant LRRK2 does not alter the proportion of peripheral immune cell subsets.

We next transplanted Rag1/R1441G double mutant mice with BM-derived from sex- and MHC-matched WT(GFP) donors. As a result of BMT, the immune system of these chimeric mice (WT(GFP) -> Rag1/R1441G) expressed *wt*LRRK2 in T- and B-cells (Fig. 1c), but their innate immune cells, as well as the CNS (and all other cells/organs that normally express LRRK2), expressed R1441G LRRK2. All BM-transplanted double mutant chimeras developed normally without any signs of Graft vs Host Disease (GvHD) and had a 0% post-BMT mortality rate. To further validate that T- and B-cells from BM transplanted double mutant mice lack human mutant LRRK2, we sorted double-positive GFP+/CD4+, GFP+/CD8+, and GFP+/CD19+ cells and analyzed human and mouse *lrrk2* mRNA expression. As expected, peripheral lymphocytes from double mutant chimeras highly express *wt* mouse *lrrk2* (Fig. 1d) demonstrating that T- and B-cells are WT donor-derived. Similar to Rag1 chimeras, the baseline antibody and cytokine profiles in the double mutant chimeras carrying a *wt* adaptive immune system were fully restored to the control WT level (Fig. 1e, f).

Since it has been demonstrated in several disease models including bacterial meningitis, autoimmune encephalomyelitis, PD, and Alzheimer’s disease, as well as in certain experimental conditions (for example, in PU.1 mice lacking all cells of myeloid and lymphoid lineages) that BM stem cells can migrate across the BBB and transdifferentiate into functional microglia^39–41,48^ or even neurons^49–51^, we next assessed whether GFP + donor cells can colonize the brain of BM-transplanted double mutant chimeras before and/or after LPS injection. Histological analysis of GFP co-localization with Iba-1 and GFAP revealed no contribution of BM-derived cells to the brain glial pool of chimeras (Supplementary Fig. 3a), further confirming that the CNS of double mutant chimeras remained “donor cell-free”, and therefore all cells in the brain contained a mutant LRRK2 genotype. In addition, double mutant chimeras were examined for histopathological changes including the loss of SNpc DA neurons (Supplementary Fig. 3b) and microglia activation where no abnormalities were observed.

### Replacement of mutant LRRK2 with *wt* form in peripheral T- and B-cells rescues LPS-induced SNpc DA neuron loss

**Fig. 2:**
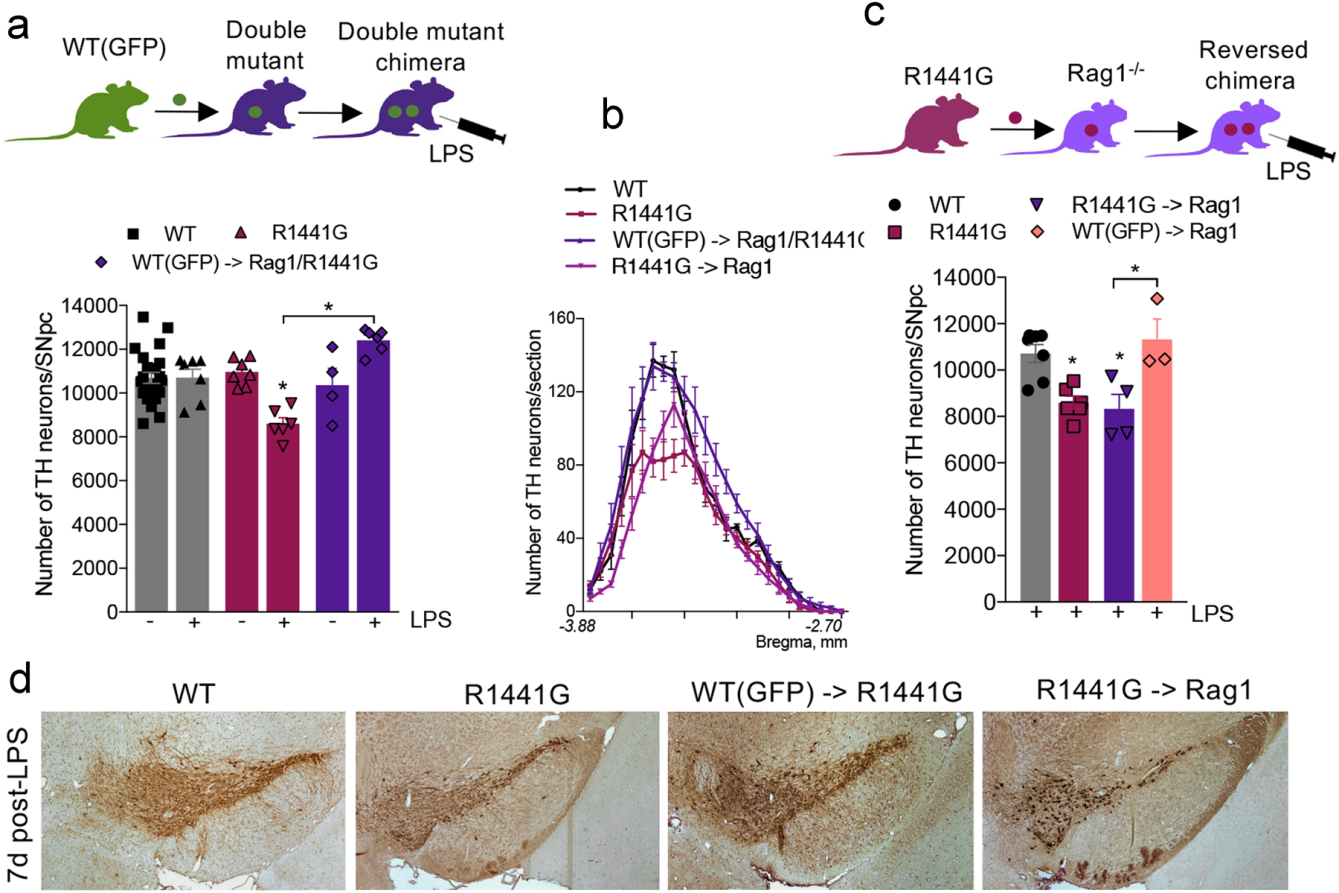
Genotype of the adaptive immune system has primacy over the genotype of the brain in regard to induction of LPS-induced neuronal loss. **a** Double mutant chimeras (WT(GFP)->Rag1/ R1441G) expressing *wt* LRRK2 in T- and B-cells show completely rescued TH cell loss compared to LPS-treated R1441G mice. Data are mean ± SEM, *n* ≥ 4. *p < 0.05 vs WT or vs group indicated on the graph. **b** Rostro-caudal gradient of DA cell loss seen in R1441G mice was restored in double mutant chimeras carrying *wt* immune cells and mimicked in reversed chimeras carrying mutant T- and B-cells. **c** Reversed chimeras expressing mutant LRRK2 only in adaptive immune cells develop SNpc TH cell loss 7 days after LPS treatment similar to LPS-treated R1441G mice. Note that LPS-treated control Rag1^−/−^ mice transplanted with GFP^+/+^ BM did not develop LPS-induced DA cell loss. Data are mean ± SEM, *n* ≥ 4. *p < 0.05 vs WT or vs group indicated on the graph. **d** Representative images of TH-positive DA neurons in the SNpc of WT, R1441G mice, and chimeras 7 days post-LPS injection. Sections are matched at the same level of the SN (Bregma −3.08–3.16 mm)^112^. Scale bar = 100 μm.

To address the question of whether mutant LRRK2 mediates its genotoxic effect through peripheral immune function, we next treated BM-transplanted double mutant chimeras carrying only *wt*LRRK2 in the adaptive immune cells with the dose of LPS previously shown to induce SNpc DA neuron loss in fully mutant R1441G mice^52^. TH-immunostaining analysis and subsequent quantification of midbrain sections revealed that SNpc DA neuron loss following LPS treatment was completely abolished in double mutant chimeras (Fig. 2a). Notably, in this experiment, LPS-induced neuronal loss in non-transplanted (e.g., immunodeficient) double mutant mice (Rag1/R1441G) could not be estimated since all mice died within 1–2 h post-LPS. This rapid death was likely attributable to a cytokine storm, as it has been shown that large numbers of naive T-cells are needed to efficiently temper the deadly TLR innate immune response at the initial phase of the infection^47^. Remarkably, we found that similar to PD patients^53,54^, inflammatory stimuli-induced SNpc DA neuron loss in R1441G mice varies significantly along the rostro-caudal axis of the SNpc; being higher in the caudal section than in the rostral one, and that this uneven pattern of DA cell loss was completely restored to the normal level in R1441G chimeras carrying *wt* immune cells (Fig. 2b, d). This further confirms the selective vulnerability of SNpc DA neurons to inflammatory conditions. Thus, our data indicate that normalization of mutant LRRK2 expression specifically in the adaptive immune cells impacts the survival of SNpc DA neurons following an exogenous inflammatory insult.

### Reversed chimeras carrying R1441G or G2019S LRRK2 in the adaptive immune system alone exhibit LPS-induced neurodegeneration

To further test our hypothesis that the peripheral adaptive immune system has primacy over the CNS innate immune system in LPS-induced neurodegeneration, we “switched” chimerism and generated mice that harbored mutations at the R1441G position of LRRK2 within the cells of the adaptive immune system, while the cells in the brain (and other cells in the body) were genotypically *wt* (Fig. 2c). We then treated these animals (R1441G ->Rag1) with the dose of LPS previously shown to induce SNpc DA neuron loss in fully mutant R1441G mice. Stereological analysis revealed that the reversed chimeras developed a significant SNpc DA neuron loss 7 days post-LPS treatment; with the same selective vulnerability of caudal SNpc DA neurons as the parental R1441G mice (Fig. 2b, c, d). Importantly, no SNpc DA neuron loss was seen in LPS-treated control Rag1^−/−^ mice transplanted with WT(GFP) BM; suggesting that the BMT procedure itself does not affect neuronal survival. To examine if this peripheral effect was specific to the R1441G mutation in LRRK2 or was common to other mutations in the LRRK2 loci, we used BMT methods to generate mice that had mutant G2019S LRRK2 in the cells of the peripheral immune system while maintaining *wt* LRRK2 in all other cells, including neurons. The G2019S mutation is situated in the LRRK2 MAPK domain that is strongly associated with a constitutive kinase activation^55^. The R1441G is located in the ROC domain of the LRRK2 gene^56–58^ but shares common pathogenic mechanisms that lead to PD^59^. Similar to fully mutant R1441G and G2019S strains, reversed G2019S ->Rag1 chimeras also develop LPS-induced neurodegeneration (Supplementary Fig. 4). These data strongly suggest that the genotype of the peripheral immune system, whether *wt* or mutant, has primacy over the brain, although it remains to be determined if the mechanisms for peripheral immune signaling are the same in both mutations.

### Inhibition of peripheral IL-6 rescues LPS-induced SNpc cell loss in R1441G LRRK2 mice

**Fig. 3:**
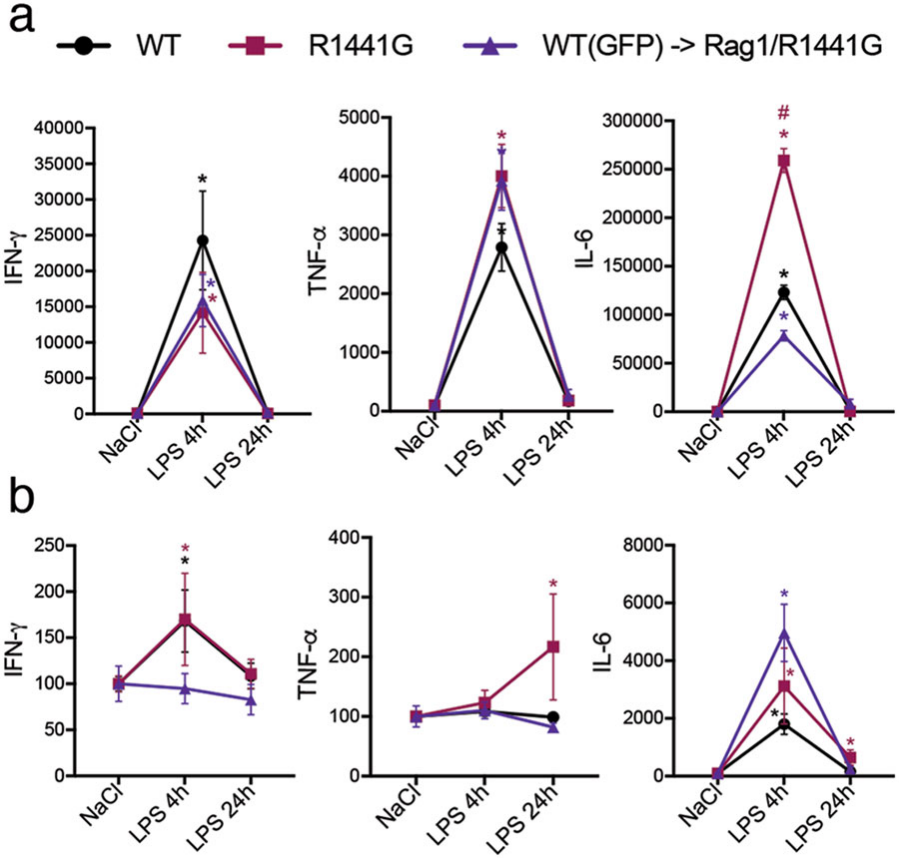
LPS-induced activation of systemic IL-6 in double mutant chimeras is back to the WT level. Cytokines profile in the serum (**a**) and SN (**b**) of WT, R1441G, and double mutant chimeras at 4–24 h after LPS injection. Data are mean ± SEM, *n* = 5–11. *p < 0.05 vs NaCl or vs group indicated on the graph.

To examine whether switching from mutant LRRK2 to *wt* in T- and B-cells resulted in a distinct inflammatory response, we next screened LPS-induced changes of predominantly pro-inflammatory cytokines in the periphery and in the brain (SN) of WT, fully mutant R1441G and rescued double mutant chimeras 24 h post-LPS. The proinflammatory cytokines examined were shown to be upregulated in LPS-treated mutant R1441G mice compared to WT, with IL-6 expression showing the greatest increase in the serum of R1441G mice 4–9 h post-LPS compared to control animals (Fig. 3a). Notably, we observed that peripheral IL-6 expression in double mutant chimeras returned to the WT level while expression of TNF-α and IFN-γ remained unchanged (Fig. 3a). Furthermore, IL-6 level in the SN of double mutant chimeras also normalized to WT level 24 h post-LPS (Fig. 3b).

**Fig. 4:**
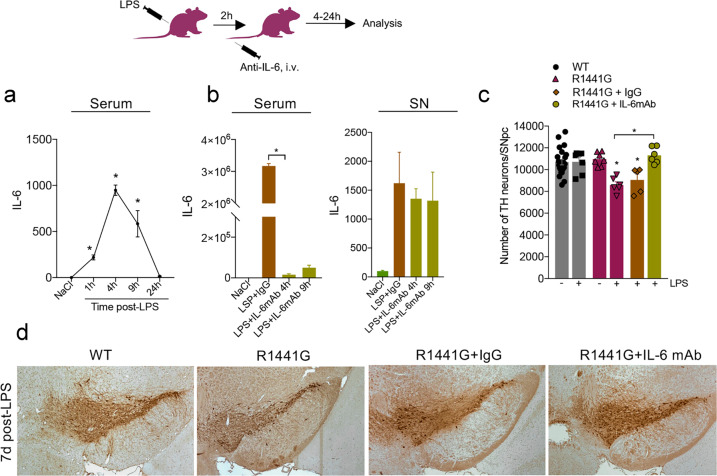
Inhibition of peripheral IL-6 rescues LPS-induced SNpc cell loss in R1441G mice. **a** Time-course of serum IL-6 secretion in R1441G mice at different time points post-LPS. Data are mean ± SEM, *n* = 5–8. *p < 0.05. **b** Anti-IL-6 antibody significantly inhibits LPS-induced IL-6 up-regulation in serum, with no change was seen in the SN. Data are mean ± SEM, *n* = 3–5. *p < 0.05. For each group, data in LPS-treated groups are expressed as the percentage of NaCl control. **c** Neutralization of peripheral IL-6 completely rescues LPS-induced SNpc DA neuron in R1441G mice. Data are mean ± SEM, *n* ≥ 4. *p < 0.05 vs WT or vs group indicated on the graph. **d** Representative images of TH-positive DA neurons in the SNpc of LPS-treated WT, R1441G, and R1441G that received either anti-IL-6 mAb or IgG. Sections are matched at the same level of the SN (Bregma −3.08–3.16 mm) (Paxinos and Franklin, 2001). Scale bar = 100 μm.

Given that IL-6 was the only one of these cytokines consistently observed to be changed in rescued chimeras with *wt* adaptive immune cells, we reasoned that modulation of peripheral IL-6 signaling in fully mutant LRRK2 mice might alter the program of CNS neurodegeneration. In order to experimentally validate this hypothesis, we treated R1441G LRRK2 mice with monoclonal anti-IL-6 antibody (2.5 mg/kg b/w; i.v. injection) or mouse IgG (control) 2 h after LPS injection. This time point (2 h post-LPS) was chosen empirically based on our finding that showed serum IL-6 levels rapidly increased (within 1–1.5 h) after the administration of LPS (Fig. 4a). R1441G mice treated with anti-IL-6 antibody had a significant reduction of serum IL-6 compared to IgG-treated control mice, while no change was seen in its levels within the SN (Fig. 4b), suggesting the modulation of IL-6 was entirely peripheral. Subsequent immunohistochemical analysis revealed that neutralization of peripheral IL-6 overproduction completely rescued the LPS-induced SNpc DA neuron loss seen in R1441G mice 7 days after treatment. No change in LPS-induced neuron loss was seen in a control cohort of LRRK2 mice treated with IgG (Fig. 4c, d). These data, thus, indicate that peripheral IL-6 signaling plays a crucial role in peripheral immune response-mediated neuronal loss in LRRK2-PD.

## Discussion

In this study, using a chimeric mouse model, we provide the evidence demonstrating that alterations in the adaptive immune system of animals carrying LRRK2 mutations can dramatically alter the development of the neuropathological phenotype. Specifically, we found that LPS-induced loss of DA neurons in the SNpc of mice overexpressing pathogenic LRRK2 mutations can be abolished by the replacement of mutant LRRK2 in T- and B-lymphocytes with the *wt* form of the protein. Importantly, we also found that harboring a genotypically *wt* CNS but possessing the R1441G or G2019S LRRK2 mutation in lymphocytes alone is sufficient to sensitize nigral DA neurons to the effects of an exogenous agent, in this case, LPS. Mechanistically, we have identified peripheral IL-6 as a key effector of the cascade of events that lead to LRRK2-mediated neurotoxicity. These results represent a major paradigm shift in our understanding of PD pathogenesis and suggest that immune dysfunction in some forms of familial PD may have primacy over the CNS as the initiating site of the disorder.

It is now apparent that the CNS, like other organs, is under regular immune surveillance, and that the prevailing neuro-immune connection is not limited to localized microglia-driven neuroinflammation inside the brain. In fact, growing evidence suggests that perturbations in both peripheral innate and adaptive immune systems have a major impact on the brain function, behavior, and the development of neurological disorders^60–63^. A number of animal studies have demonstrated that changes in the peripheral immune repertoire can significantly influence cognition and neuronal loss in both injury and neurodegenerative disease models. Immunodeficient SCID mice have been shown to have impaired cognitive and emotional behaviors were attributed specifically to CD4 + T cells^60,64–66^. Other studies have shown that peripheral immune activation by the TLR3 agonist Poly(I:C) causes synapse loss which is mediated by CX3CR1^high^ monocytes via TNF-α-dependent mechanisms^67^. Moreover, IL-4 producing Th2 cells exert beneficial effects on neurons upon traumatic CNS injury^61^. Multiple reports have also implicated a role for peripherally derived neutrophils and T-cells in Alzheimer disease pathogenesis^46,68,69^. The recent discovery of a rich diversity of peripherally originated immune cells in both mouse and human meninges^70–72^, a location where these immune cells form a direct interface with the brain parenchyma, suggests that any alterations in the peripheral or meningeal immunity can profoundly influence CNS homeostasis and give rise to neurological disorders. Our group has previously demonstrated that neuroinflammation and neuronal loss in LRRK2-PD were not directly associated with the recruitment of peripheral T-cells or monocytes into the brain parenchyma, nor was it mediated through microglial LRRK2^52^. Similar results have been observed ex vivo where neither LPS nor priming with α-synuclein PFFs increased LRRK2 protein expression in murine microglia^73^, further confirming that LRRK2 levels in the brain resident immune cells may not have a direct effect on inflammation-mediated neuronal loss.

In contrast to brain microglia, systemically circulating monocytes, neutrophils, T- and B- cells display a high level of LRRK2 and its expression is induced upon immune activation^28,29,74–76^. An increased induction of LRRK2 protein was observed in neutrophils and CD8+ T cells from PD patients following stimulation with IFN-γ ^32,77^, while LRRK2 mRNA was upregulated in B-cells from patients with systemic lupus erythematosus^78^. Elevated LRRK2 levels were also found in CD14+ and CD16+ monocyte subsets of PD patients^79^. In regard to adaptive immunity, it has been shown that *wt* LRRK2 inhibits the nuclear translocation of NFAT1 (NFATc2), a master regulator of transcriptional activation in T-cells^80,81^, and negatively regulates antigen processing during phagosome maturation^82^. This suggests that LRRK2 is involved in regulating the activation status and effector functions in multiple kinds of immune cells. These findings led us to postulate that pathogenic PD mutations in the LRRK2 gene might contribute to dysregulated molecular pathways in peripheral immune cells, which in turn act as a driving force to influence the neuronal loss. Although studies examining the microglia-mediated neuroinflammation and neuronal injury in animal models of PD are well documented (for review, see^83,84^), there was currently a lack of direct evidence that peripheral immunity is critical in the selective death of SNpc DA neurons in PD. We, therefore, in the current study prove that under certain inflammatory conditions, DA neuron loss in the SNpc completely depends on the genotype of the adaptive immune cells. We conclude this based on our results showing that T- and B-cells carrying a R1441G or G2019S mutation induce neurodegeneration in the normal *wt* brain, while lymphocytes expressing *wt* LRRK2 protect the SNpc against LPS-induced neuron loss in animals expressing mutant LRRK2 in their brain. Given that we observed no invading lymphocytes or monocytes in the brain parenchyma of LRRK2 mice- even after LPS treatment- we suggest that altered peripheral immune cells might mediate their effect either directly via systemic cytokine signals capable of crossing the BBB (through an active transport system) or from the meningeal space where they can release soluble mediators that, through paracrine signaling, affect neurons and other CNS cells. When examining cytokine expression in double mutant chimeras expressing *wt* LRRK2 in T- and B-cells, we found a significant downregulation of LPS-induced IL-6 secretion compared to that of fully mutant R1441G LRRK2 mice; and that attenuation of peripheral IL-6 signaling had a beneficial effect on the survival of SNpc DA neurons in LRRK2 mutants. However, the lack of central IL-6 down-regulation upon peripheral neutralization with antibody along with the studies showing that systemically circulating IL-6 has only limited ability to enter the brain’s parenchyma (0.2% of peripheral dose)^85^, suggests that peripherally derived IL-6 is unlikely to directly act on its CNS targets. This observation raises two important questions: (i) what is the cellular source of dysregulated systemic cytokines, specifically IL-6, in LRRK2 mice? and (ii) do these cytokines act on the CNS indirectly by inducing release of other soluble mediators from the immune cells located closest to the brain, in the meninges?

IL-6 was originally isolated from macrophages and T-cells as a B-cell growth factor which plays an important role in antibody production and class switching^86,87^. However, recent studies have suggested that a significant amount of IL-6 (in some cases much higher than in macrophages) can be produced by B-cells in response to LPS^88–90^. Given these features of IL-6, along with our previous observation of significant upregulation of mutant LRRK2 expression in CD19+ B-cells upon in vivo LPS treatment^52^, it is possible that this sub-population of immune cells is a major contributor to IL-6 overproduction. In fact, ongoing experiments in our lab suggest that a specific deletion of mutant LRRK2 in CD19+ B-cells might have a neuroprotective effect on SNpc DA neurons following LPS administration. Since the meninges are highly populated by many different bloodstream-derived immune cell including B-cells, each of which can release various cytokines into the CSF^65,66,70,91,92^, it is plausible to speculate that mutant LRRK2 might mediate its neurotoxic effect via disrupted meningeal-immune cell trafficking and cytokine signaling.

Two recent studies strongly implicate meningeal lymphatic dysfunction in the pathogenesis of PD. Animal experiments showed that blocking extracranial lymphatic drainage markedly exacerbates accumulation of α-synuclein in the brain of A53T mice and subsequently leads to extensive reactive astrogliosis^93^. Second, using dynamic contrast-enhanced MRI, it was found that patients with idiopathic PD exhibited significantly reduced flow through the meningeal lymphatic vessels along the superior sagittal sinus and sigmoid sinus, as well as a notable delay in deep cervical lymph node perfusion^94^. Thus, in future studies, it will be very important to determine whether pathogenic mutations in LRRK2 result in the accumulation of peripherally-derived B-cells in the meninges, and whether interference with the bloodstream -> meninges traffic and/or meningeal cytokine production would influence neuronal loss and the development of a parkinsonian neuropathological phenotype.

Our results suggesting that peripheral IL-6 is critical to inflammation-mediated LPS-induced neuronal death are also interesting in light of a recent in vitro study showing that *wt*LRRK2 modulates the production of TNF-α and IL-6 in α-synuclein-exposed mouse microglia; this effect being mediated through increased nuclear translocation of NFATc2^81^. Previously, NFATc2, which is known to play a critical role in T- and B-cell activation and differentiation, was shown to transcriptionally regulate the expression of IL-6 and IL-6R in different types of immune cells^95–99^. *Wt*LRRK2 was suggested as a negative regulator of NFATc2 based on its interactions with noncoding RNA repressor of NFATc2 to inhibit nuclear translocation^80^, and it was proposed that selective blocking NFATc2 phosphorylation by LRRK2 inhibition may prevent toxic inflammation. Further in vivo studies would be incredibly valuable to determine whether the aberrant inflammatory response and SNpc DA neuron loss are mediated via dysregulation of the LRRK2-NFATc2 pathway in peripheral and/or meningeal immune cell subsets. It is also important to note that IL-6 represents a reasonable target since its concentration was shown to be increased in the nigrostriatal region and CSF of idiopathic PD patients as well as bi-allelic PARKIN carriers^100,101^. Additionally, it will be crucial to examine whether the IL-6 mAb-mediated neuroprotection sustains for a significant period of time (months).

It is also worth noting that the neutrophil-to-lymphocyte ratio in blood is significantly higher in PD patients compared to healthy controls^102^. Given that LRRK2-expressing neutrophils^76^ are the most powerful source of ROS, these cells may play an important role as signaling messengers in the immune system such that abnormal oxidative stress levels in activated circulating neutrophils might also contribute to a dysregulated immune response. In addition, neutrophils are also present in the CSF and meninges, and express high level of Rab10 which is known to regulate phagosomal recycling^103^, and future research will address this questions.

In addition to understanding of the mechanisms by which LRRK2 may exert its pathogenesis in the development of PD, our studies also have implications for therapeutic approaches to prevent or slow the progression of this disease. Our findings support the hypothesis that signals emanating from the peripheral adaptive immune system are the initiator of the cascade that eventually leads to development of PD pathology. A number of compounds that block the effects induced by mutations in LRRK2 are currently in development, with a number of these in early Phase II clinical trials. One of the issues facing development of this class of drugs is the need to find a BBB-penetrant drug that can penetrate into the brain in sufficient quantities to inhibit the neurotoxic effect of mutant LRRK2. This often requires higher doses of the drug than would be necessary to inhibit the enzyme in the periphery alone where cell accessibility is significantly improved. Our studies, in respect to LRRK2 carriers, suggest that CNS-penetrant forms of LRRK2 inhibitors might not be necessary; and that if treatment is started early enough in identified mutant LRRK2 carriers, the abnormal inflammatory signals that originate in the periphery could be normalized; which we hypothesize will reduce or eliminate the initiation of a pathogenic cascade in the brain. Given that these compounds exist, this mechanism for normalizing abnormal LRRK2 signaling in the periphery can be empirically tested. Additionally, it will be useful to examine if the primacy of the immune system in disease initiation that we describe in this study is specific to the LRRK2 mutations or if this a commonality to other familial PD genes that are shown to be “risk factors” rather than direct drivers of the disorder.

## Methods

### Animals and generation of double-mutant mice

One to five months old WT (C57BL/6J), C57BL/6J-Tg(LRRK2*R1441G)3IMjff/J, C57BL/6-Tg(UBC-GFP)30Scha/J, B6.129S7-Rag1^tm1Mom^/J mice (all Jackson Laboratory, Bar Harbor, ME) and C57BL/6-Lrrk2tm4.1Arte (Taconic, NY) were used in this study. In all experiments, age- and background strain-matched mice were used as controls. Equal number of males and females were used in the study since, in our hands, no differences in the neuropathological phenotype (the number of TH neurons) were observed between two sexes both at baseline and after LPS exposure^37^ (data not shown). C57BL/6J-Tg(LRRK2*R1441G)3IMjff/J mice (referred to hereinafter as R1441G) overexpress a human mutant LRRK2 (directed by the human LRRK2 promoter on the BAC transgene) in the brain (SN and striatum) and PBMCs at an approximately two- and four-fold greater levels than the endogenous mouse LRRK2, respectively^52^. A human LRRK2 transgene insertion site was previously located in mChr 1^52^. The C57BL/6-Lrrk2tm4.1Arte mouse strain (referred to hereinafter as G2019S) is a constitutive KI of the mouse LRRK2 gene. The human G2019S gain-of-kinase activity mutation was introduced into exon 41 of the mouse LRRK2 gene using C57BL/6 J BAC–DNA. KI mice were crossed to WT C57BL/6NTac every fourth generation to guard against genetic drift. Similar to the previously described LRRK2 KI and transgenic models, this strain does not develop age-related loss of DA neurons^104^.

In order to generate double mutant mice, homozygous B6.129S7-Rag1^tm1Mom^/J mice (referred to hereinafter as Rag1^−/−^) mice were crossed with transgenic R1441G mice to first generate heterozygous Rag1^-/+^/R1441G^wt/tg^ mice which were next intercrossed to generate homozygous double mutant Rag1^−/−^/R1441G^tg/tg^ mice (referred to hereinafter as Rag1/R1441G). Genotypes of all strains were determined by PCR of tail DNA using protocols from Jackson Laboratory. All experimental procedures were performed under pathogen-free conditions. All animals were housed within the vivarium at Thomas Jefferson University and maintained on a 12:12 h (h) light/dark cycle with ad libitum food and water. All of the experimental procedures in the animals were performed in accordance with the NIH Guide for the Care and Use of Laboratory Animals and all protocols were approved by the Thomas Jefferson University IACUC (Protocol 1892). Experiments were carried out in accordance with The Code of Ethics of the World Medical Association (Declaration of Helsinki) for animal experiments.

### Bone marrow isolation and transplantation

In order to generate chimeric mice, 5-6 week old recipient Rag1^−/−^ mice^44^ or double mutant Rag1/R1441G mice lacking T- and B-cells were injected i.v. with bone marrow (BM) cells derived from donor sex- and MHC-matched homozygous C57BL/6-Tg(UBC-GFP)30Scha/J (referred to hereinafter as WT(GFP) mice). BM cells were isolated from the femurs and tibias of euthanized 8–15 wk old donor mice, treated with RBC lysis buffer, washed, and 20 × 10^6^ cells in 200 ul of sterile saline were immediately injected into the lateral tail vein. Only freshly isolated BM cells were used in the study. Donor WT(GFP) mice have a ubiquitin promoter-driven GFP transgene expressed throughout all hematopoietic cell subsets and developmental stages and were generated directly in the C57BL/6 strain making this strain ideal for cell-transfer experiments into C57BL/6 recipients^105^. To generate “reversed” chimeras (referred to hereinafter as R1441G ->Rag1 or G2019S ->Rag1), immunodeficient Rag1^−/−^ recipients were transplanted with BM-derived from homozygous R1441G or G2019S donor mice (8–15 wk old; 20 × 10^6^ cells). All resulted chimeras (WT(GFP) -> Rag1, WT(GFP) -> Rag1/R1441G, R1441G ->Rag1 and G2019S ->Rag1) were monitored 2 times/week first 2 weeks post-BMT for any signs of rejection and once per week thereafter. NaCl- or LPS-treated 4-5 months old WT (C57BL/6J), Rag1^−/−^, R1441G, and G2019S mice were used as controls for chimeric strains.

### LPS and neutralizing antibody administration

Eight weeks post-BMT, chimeras were injected i.p. with 5 mg/kg LPS (*E.coli* serotype O111:B4; Sigma–Aldrich MO, USA). Immediately after LPS injection, mice were placed on warming pads in order to prevent hypothermia and reduce mortality^106,107^. Mice were monitored daily for the next 7 days. All experimental procedures were performed under pathogen-free conditions. In cytokine neutralization experiments, 2 h post-LPS mice received either anti-IL-6 monoclonal neutralizing antibodies (2.5 mg/kg body weight, i.v.; Thermo Fisher, USA) or control IgG (Thermo Fisher, USA) in 200 μl of sterile saline via injection into the lateral tail vein.

### Peripheral blood mononuclear cells separation

Blood was collected from the heart of deeply anesthetized mice into 10% EDTA-treated vacuum tubes, mixed 1:1 with sterile PBS, and separated in lymphocytes separation medium (Corning, NY USA). Samples were centrifuged for 30 min at 400 g at RT and then the leukocytes “white” layer was carefully aspirated and placed into sterile tubes. Cells were washed with sterile PBS, centrifuged for 10 min at 200 *g* at RT, and then immediately used for Flow Cytometry or FACS.

### Flow cytometry and FACS

To characterize the immune phenotype of double mutant mice and verify the reconstitution of T- and B-lymphocyte populations in BM-transplanted mice, spleen and PBMCs were collected and after single-cell suspension was obtained, stained with antibodies directed against CD4, CD8, CD19, CD11b, and DAPI (all from BD Biosciences, San Diego CA). After eliminating debris/dead cells (DAPI-positive) and doublet cells by forward- and side-scatter, cells were gated based on CD4 (PerCP-Cy5.5), CD8 (PE-Cy7), CD19 (APC-Cy7), CD11b (Alexa700) and/or GFP fluorescent intensity. A small portion of the cells was incubated without antibodies to gate cells according to their light scattering characteristics, and another small portion of the cells was stained with isotype-matched control reagents to set the maximum threshold for non-specific binding. Data were acquired using a FACSCalibur flow cytometer (BD Biosciences, San Diego CA) and analyzed with CellQuest Pro (BD Biosciences, San Jose, CA) software. The final flow data in control mice and double mutants were represented as the percentage of each sub-population (CD4+, CD8+, CD19+) to the total number of live cells analyzed. The final data in BM-transplanted chimeras were represented as the percentage of GFP + cells to the total number of live cells. Homozygous GFP^+/+^ and Rag1^−/−^ mice were used as positive and negative controls, respectively. For FACS, PBMCs were isolated from double mutant transplanted chimeras (WT(GFP) -> Rag1/R1441G) and sorted for GFP^+^/CD4^+^, GFP^+^/CD8^+^, GFP^+^/CD19^+^ as well as for GFP-negative signal using a FACSAria cell sorter. The viability of sorted cell populations was always at least 95%. After sorting, cells were washed with sterile PBS, centrifuged, and stored at −80 °C until assayed.

### RT-qPCR

FACS-sorted cells were processed to obtain RNA in accordance with the protocol outlined in PureLink® RNA Mini Kit or ARCTURUS® PicoPure® RNA isolation Kit (Thermo Fisher Scientific, MA, USA). The RNA samples were treated with DNase (Thermo Fisher Scientific, MA, USA) and total RNA was quantified using a NanoDrop 8000 (Thermo Fisher Scientific, MA, USA). Isolated RNA was converted to cDNA using a High-Capacity RNA to cDNA kit (Applied Biosystems, CA, USA) according to manufacturer instructions. This cDNA was subsequently used for RT-qPCR analysis using specific validated primers (Taqman® assays) obtained from Life Technologies. Reactions were performed using TaqMan Gene Expression Master Mix or TaqMan® Fast Advanced Master Mix in a 7300 Real-Time PCR System or 7500 Fast Real-Time PCR System (Applied Biosystems, USA), respectively. β-actin gene was used as the normalizing/housekeeping gene. Gene expression was expressed as 2^−ΔΔCt^ denoting fold-change in mRNA levels for each gene.

### Luminex and ProcartaPlex assays

For cytokine assays, blood was collected into EDTA-treated tubes. Serum was separated by centrifugation and stored at −20 °C until assay. Brain tissues were mechanically homogenized in Bioplex cell lysis buffer containing factors 1 and 2 (Bio-Rad, CA, USA) and centrifuged at 4500 × *g*. The total protein concentration of each sample was determined using the BCA assay (Pierce, IL, USA), with bovine serum albumin as a standard, according to the manufacturer’s protocol. Individual vials of cytokines/chemokines beads were sonicated, vortexed and then mixed with assay buffer (Milliplex Map Kit, MCYTOMAG-70K, Millipore, MA, USA). Working standards were made by diluting the stock concentration (10,000 pg/ml) in assay buffer. Brain and serum samples were added in equal volumes (25 μl) into the wells (96-well plate) containing 25 μl of assay buffer and 25 μl of cytokines mixed beads and plate was incubated overnight at +4 °C. Then plate was washed and incubated following: (i) with detection antibodies, (ii) Streptavidin-Phycoerythrin, (iii) sheath fluid. The plates were run on a Luminex 200^TM^ according to manufacturer’s recommended procedures and the data analyzed using BioPlex Manager 4.1 software (Bio-Rad, Hercules, CA). All samples were run in duplicates. Data were expressed as pg/mg total protein. Final data in LPS-treated groups are expressed as the percentage of NaCl control.

The antibody assay was performed on serum using Mouse Antibody Isotyping 7-Plex ProcartaPlex Panel (Invitrogen, Waltham, MA) measuring the antibody isotypes IgA, IgE, IgG1, IgG2a, IgG2b, IgG3, and IgM. The antibody assay was performed according to the manufacturer’s instructions. The plates were read on Luminex 200^TM^ according to manufactures recommended procedures and the data analyzed using BioPlex Manager 4.1 software.

### Immunohistochemistry

Mice were intracardially perfused with 1× phosphate-buffered saline (PBS), pH 7.4. followed by 3% paraformaldehyde in PBS, pH 7.4. Each brain was dissected out of the skull following perfusion and post-fixed overnight in fresh fixative. Brains were then dehydrated through a graded series of ethanols, defatted in mixed xylenes and embedded in paraffin (Paraplast-Xtra™, Fisher Scientific), and then blocked and cut in the coronal plane and serially-sectioned at 10 µm. Every section from the rostral hippocampus to the anterior aspects of the cerebellar-midbrain junction was saved and mounted, five sections per slide, onto Superfrost-Plus slides (Fisher Scientific, MA, USA). Every other slide (first series) was double labeled for TH (1:250; mouse monoclonal, T1299, Sigma-Aldrich, MO, USA) and ionized calcium-binding adapter molecule 1 (Iba-1) (1:200; rabbit polyclonal, 019–19741, Wako), VA, USA) as described previously^108^. The double labeling was carried out using a two-color DAB protocol. In some experiments, a second series of sections were labeled for T-cells (CD3, 1:300; Armenian hamster, 14-0031-82, Santa Cruz Biotechnology, TX, USA). All sections were then counterstained with a Nissl stain (Neutral Red or cresyl violet).

To examine whether GFP-positive donor BM cells colonize the CNS and differentiate into microglia and/or astrocytes in non-treated and LPS-treated chimeras, 10um coronal brain sections were stained with Iba-1 (1:500; goat polyclonal, ab5076, Abcam, USA) or GFAP (1:250; mouse monoclonal, G3893, Sigma, USA). Then sections were washed and incubated with anti-goat or anti-mouse Alexa Fluor 633 antibodies (Life Technologies, NY, USA) and analyzed for co-expression of GFP in Iba-1^+^ and GFAP^+^ cells. Sections incubated without primary antibody were used as a negative control. Iba-1-stained spleen sections from double mutant chimeras were used as a positive control for GFP + donor cell engraftment.

### Microscopy and model-based stereology

The total number of SNpc TH-positive DA neurons as well as Iba-1-positive microglial cells was estimated using model-based stereology^109,110^. On average, forty sections per SNpc were analyzed. TH-positive or Iba-1-positive cells were counted as present within a section if they exhibited DAB (TH) or VIP (Iba-1) reaction product in the cytoplasm and there was a clear and complete nucleus present. For DA neurons, we also noted the presence of TH-, Nissl + DA neurons, but these were rarely identified. Microglia were also quantified. These cells were deemed as “resting” if it contained a small oval Iba-1-positive cell body that averaged three microns or less in diameter with long slender processes, and as “activated” when the cell body was increased in size compared to resting microglia and had an irregular shape with shorter and had thickened processes^111^. Double-stained GFP/Iba-1 and GFP/GFAP sections were examined with a fluorescent microscope (Olympus BX51, Olympus Corporation, Tokyo, Japan) under 10× and 20× objectives.

### Statistical analysis

All data are represented as mean ± SEM. Statistical analysis was performed using the GraphPad Prism^®^ version 4.03 software. Differences between groups were determined by t-test (2 groups) or one-way ANOVA (more than two groups). If overall statistical significance was found using ANOVA, post hoc comparisons (Tukey) were performed.

### Reporting summary

Further information on research design is available in the Nature Research Reporting Summary linked to this article.

## Supplementary information

Supplemental Data

Reporting Summary

## Data Availability

There are no data in this paper that can be submitted to a data repository. Data as reported in this paper show each data point and no data have been excluded from the analysis.
