# Supplementary material for: Mutant LRRK2 in lymphocytes regulates neurodegeneration via IL-6 in an inflammatory model of Parkinson’s disease
Source: NPJ Parkinsons Dis. 2022 Mar 15;8:24. doi: 10.1038/s41531-022-00289-9 (PMC8924242; doi:10.1038/s41531-022-00289-9)
Supplement: Supplementary file 1 — Supplemental Data [file 41531_2022_289_MOESM1_ESM.pdf]

Mutant LRRK2 in lymphocytes regulates neurodegeneration via IL-6 in an inflammatory model of Parkinson's disease

Elena Kozina[1], Matthew Byrne[1], Richard Jay Smeyne[1*]

[1]Department of Neurosciences, Jefferson Hospital for Neuroscience, Thomas Jefferson University, 900 Walnut St, Philadelphia PA 19107.

Running title: Immune system primacy in LRRK2-mediated Parkinson's disease

Keywords: LRRK2, Parkinson's disease, B cells, T cells, immune system, bone marrow transplant

[*]Corresponding author.
Richard Jay Smeyne, Ph.D.
Department of Neuroscience
Jefferson Comprehensive Parkinson's Disease Center
Thomas Jefferson University
900 Walnut Street
Philadelphia PA 19107
215-955-8634 (office)
ORCHD ID: 0000-0002-8459-5472
Email: Richard.smeyne@jefferson.edu

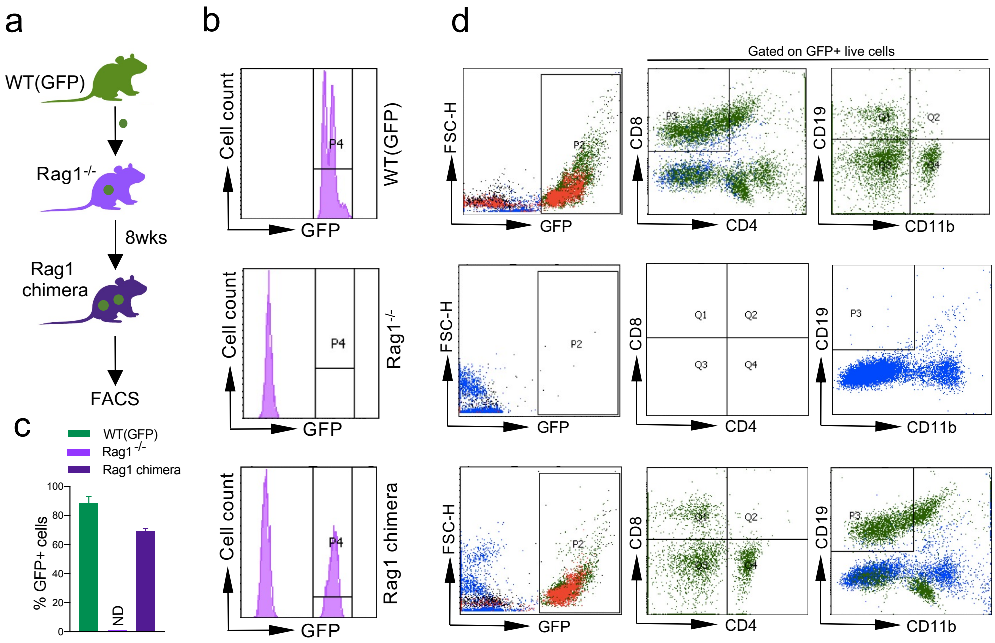

Supplementary Figure 1. Peripheral blood lymphoid chimerism in Rag1$^{-/-}$ mice 8 weeks post-BM transplantation. (a) Schematic representation of BMT experiment. Recipient Rag1-/- mice lacking B- and T-cells were injected with donor GFP$^{+/+}$ BM cells and analyzed for chimerism 8 weeks post-BMT. (b) Representative flow cytometry analysis of the frequency of donor-derived GFP+ cells in reconstituted chimeras. Donor GFP$^{+/+}$ and non-transplanted Rag1$^{-/-}$ mice were served as positive and negative controls, respectively. (c) Quantification of the percentage of GFP+ cells (% of total alive cells) in the blood of transplanted mice shows sufficient reconstitution level of donor cells. Data are mean ± SEM, n=3 for each group. (d) Gating strategy for FACS analysis of PBMCs from BM-transplanted chimera mice. After eliminating debris/dead cells and doublet cells by forward- and side-scatter, cells were gated based on CD4 (PerCP-Cy5.5), CD8 (PE-Cy7), CD19 (APC-Cy7), CD11b (Alexa700) fluorescent intensity. Positive and negative gates were drawn based on the intensity of an unstained control sample.

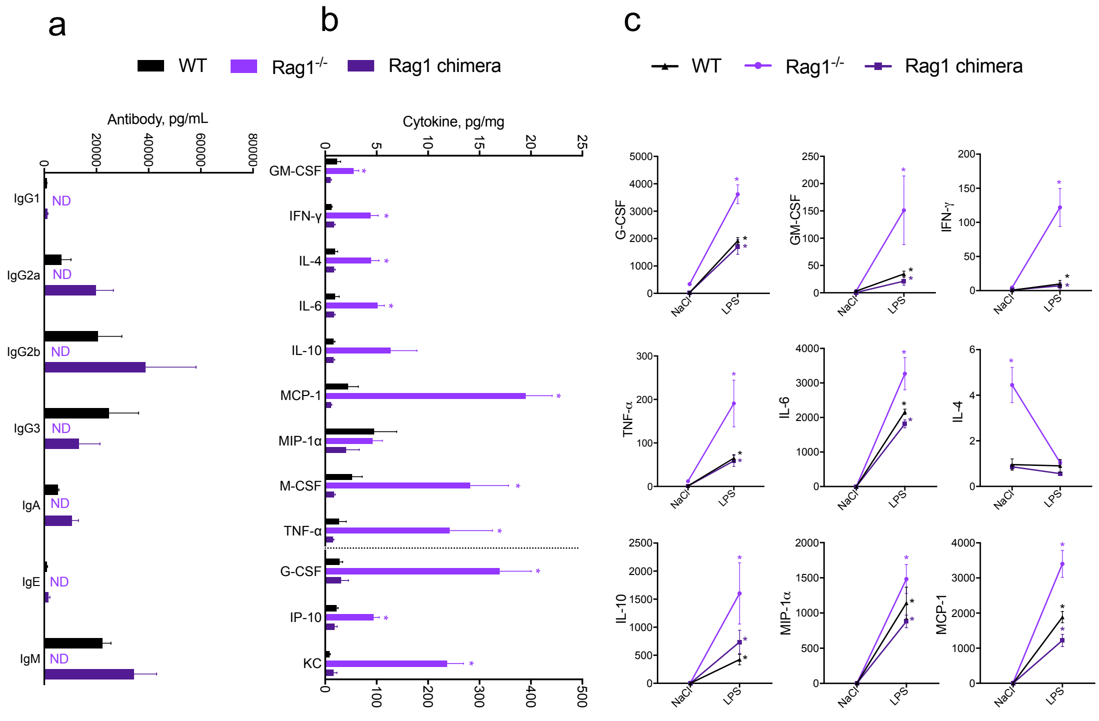

Supplementary Figure 2. Rescued immunodeficiency in Rag1 BM-transplanted chimeras. (a) Antibody deficiency seen in Rag1$^{-/-}$ recipient mice is completely restored in transplanted chimeras. (b) Baseline serum cytokine expression and LPS-induced cytokine response (c) are back to WT level in BM-reconstituted chimeras compared to non-transplanted Rag1$^{-/-}$ mice. Data are mean ± SEM, n=6 for each group. *p<0.05 vs. WT controls in B; *p<0.05 vs. NaCl controls in (c). ND, not detectable.

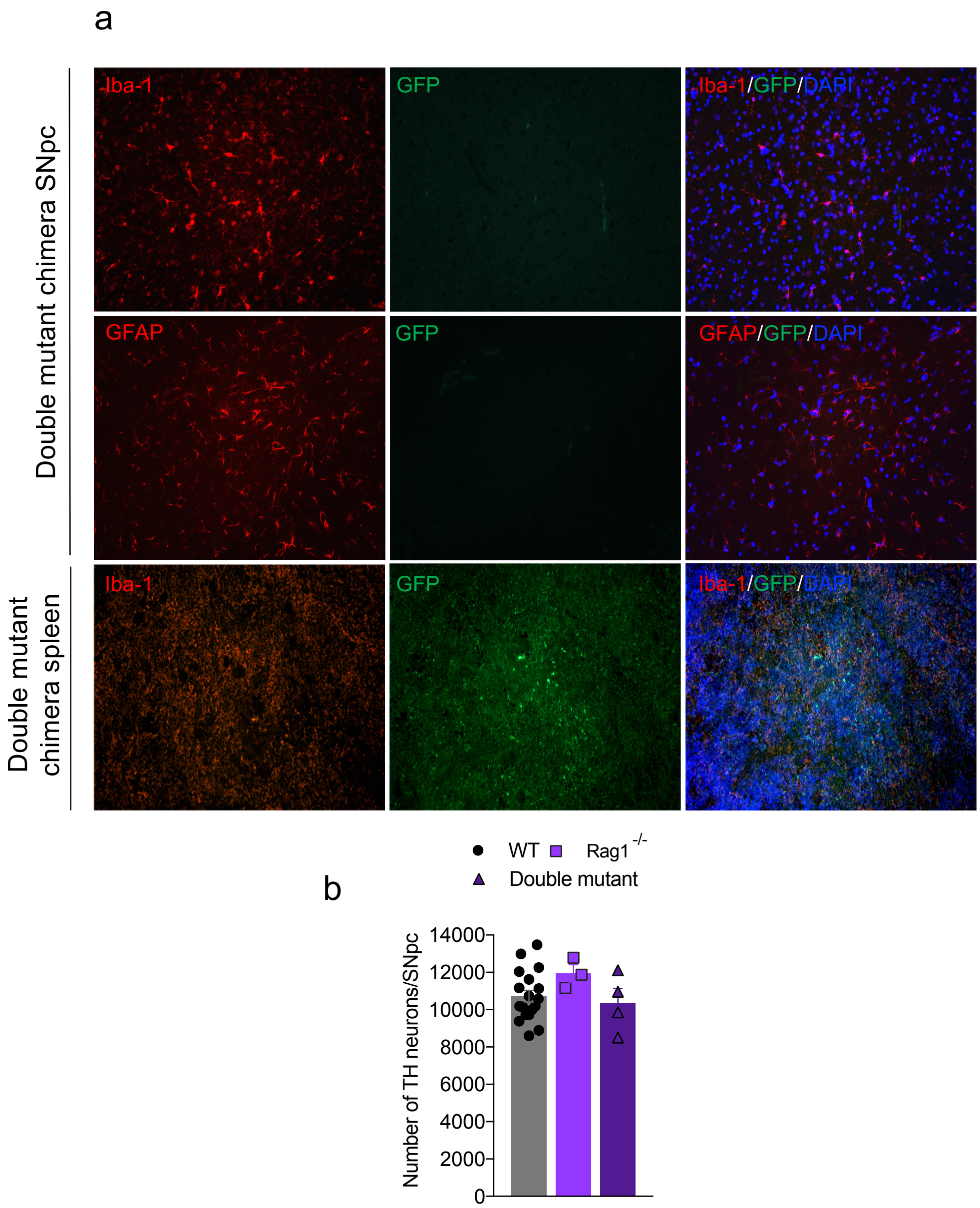

Supplementary Figure 3. Double mutant chimera's CNS remains of host origin and does not display any histopathological changes. (a) Donor-derived bone marrow cells do not colonize the brains of double mutant chimeras. Immunohistochemistry of chimeric brains shows Iba-1+/GFP- and GFAP+/GFP- cells in the SN of non-treated (not shown) and LPS-treated (a) double mutant chimeric mice 8-10 weeks after BMT. Bars = 200 mm. Iba-1 stained spleen sections from double mutant chimeras were used as a positive control of peripheral engraftment. (b) Lack of DA cell loss in the SNpc of non-treated Rag1 and double mutant chimeras. Data are mean ± SEM, n≥3 for each group.

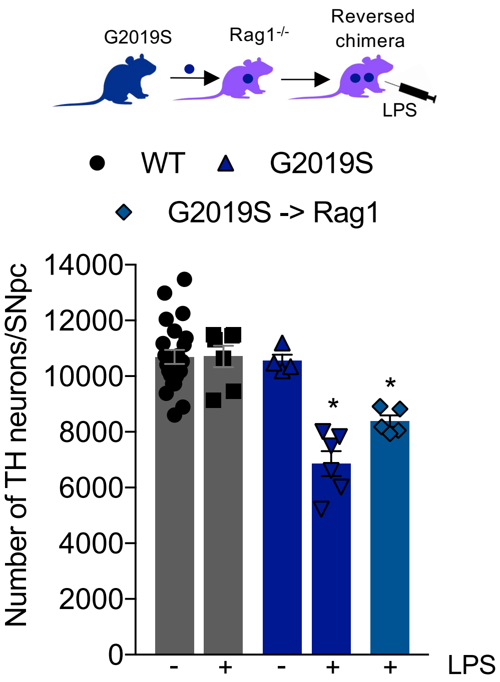

Supplementary Figure 4. Reversed chimeras expressing mutant G2019S LRRK2 in adaptive immune cells only also develop SNpc TH cell loss 7 days after LPS treatment similar to LPS-treated parental G2019S mice. Data are mean ± SEM, n≥5. *p<0.05 vs non-treated G2019S.
