# Supplementary material for: Mutant LRRK2 in lymphocytes regulates neurodegeneration via IL-6 in an inflammatory model of Parkinson’s disease
Source: NPJ Parkinsons Dis. 2022 Mar 15;8:24. doi: 10.1038/s41531-022-00289-9 (PMC8924242; doi:10.1038/s41531-022-00289-9)
Supplement: Supplementary file 2 — Reporting Summary [file 41531_2022_289_MOESM2_ESM.pdf]

nature portfolio

Corresponding author(s): Richard Smeyne, PhD

Last updated by author(s): Jan 25, 2022

Reporting Summary

Nature Portfolio wishes to improve the reproducibility of the work that we publish. This form provides structure for consistency and transparency in reporting. For further information on Nature Portfolio policies, see our Editorial Policies and the Editorial Policy Checklist.

Statistics

For all statistical analyses, confirm that the following items are present in the figure legend, table legend, main text, or Methods section.

n/a	Confirmed	
☐	☒	The exact sample size (*n*) for each experimental group/condition, given as a discrete number and unit of measurement
☐	☒	A statement on whether measurements were taken from distinct samples or whether the same sample was measured repeatedly
☐	☒	The statistical test(s) used AND whether they are one- or two-sided<br>*Only common tests should be described solely by name; describe more complex techniques in the Methods section.*
☒	☐	A description of all covariates tested
☒	☐	A description of any assumptions or corrections, such as tests of normality and adjustment for multiple comparisons
☐	☒	A full description of the statistical parameters including central tendency (e.g. means) or other basic estimates (e.g. regression coefficient) AND variation (e.g. standard deviation) or associated estimates of uncertainty (e.g. confidence intervals)
☐	☒	For null hypothesis testing, the test statistic (e.g. *F*, *t*, *r*) with confidence intervals, effect sizes, degrees of freedom and *P* value noted<br>*Give P values as exact values whenever suitable.*
☒	☐	For Bayesian analysis, information on the choice of priors and Markov chain Monte Carlo settings
☒	☐	For hierarchical and complex designs, identification of the appropriate level for tests and full reporting of outcomes
☒	☐	Estimates of effect sizes (e.g. Cohen's *d*, Pearson's *r*), indicating how they were calculated

Our web collection on statistics for biologists contains articles on many of the points above.

Software and code

Policy information about availability of computer code

Data collection	Stereological analysis of SNpc DA neuron number and microglia counts were performed using StereoInvestigator (MBF Biosciences).
Data analysis	Statistical analyses were generated using Prism 9.3 (GraphPad Software, LLC.)

For manuscripts utilizing custom algorithms or software that are central to the research but not yet described in published literature, software must be made available to editors and reviewers. We strongly encourage code deposition in a community repository (e.g. GitHub). See the Nature Portfolio guidelines for submitting code & software for further information.

Data

Policy information about availability of data

All manuscripts must include a data availability statement. This statement should provide the following information, where applicable:

- Accession codes, unique identifiers, or web links for publicly available datasets
- A description of any restrictions on data availability
- For clinical datasets or third party data, please ensure that the statement adheres to our policy

There is no data in this paper that can be submitted to a data repository. Data as reported in this paper show each data point and no data has been excluded from the analysis. All data will be made available upon request.

Field-specific reporting

Please select the one below that is the best fit for your research. If you are not sure, read the appropriate sections before making your selection.

☒ Life sciences ☐ Behavioural & social sciences ☐ Ecological, evolutionary & environmental sciences

For a reference copy of the document with all sections, see nature.com/documents/nr-reporting-summary-flat.pdf

Life sciences study design

All studies must disclose on these points even when the disclosure is negative.

Sample size	Sample size was determined based on expected data distribution of effects of toxicant effects that have been examined in over 250 animals. These parameters were used in a Power analysis (G*Power).
Data exclusions	No data was excluded.
Replication	All animals are unique to that no duplication is possible as a single "n". However, in biochemical experiments (cytokine, all samples were run in triplicate and the data point was an average of these 3 points).
Randomization	Animals were randomly assigned to experimental groups based on a "next up" strategy; using consecutive numbering. In all groups, we insured that not all animals came from a single litter.
Blinding	All stereology was performed by investigators blinded to conditions.

Reporting for specific materials, systems and methods

We require information from authors about some types of materials, experimental systems and methods used in many studies. Here, indicate whether each material, system or method listed is relevant to your study. If you are not sure if a list item applies to your research, read the appropriate section before selecting a response.

Materials & experimental systems

n/a	Involved in the study	
☐	☒	Antibodies
☒	☐	Eukaryotic cell lines
☒	☐	Palaeontology and archaeology
☐	☒	Animals and other organisms
☒	☐	Human research participants
☒	☐	Clinical data
☒	☐	Dual use research of concern

Methods

n/a	Involved in the study	
☒	☐	ChIP-seq
☐	☒	Flow cytometry
☒	☐	MRI-based neuroimaging

Antibodies

Antibodies used	All information in detail is in methods section
Validation	validation based on manufacturers website and previous use of the antibodies

Animals and other organisms

Policy information about studies involving animals; ARRIVE guidelines recommended for reporting animal research

Laboratory animals	All information in detail is in methods section
Wild animals	n/A
Field-collected samples	N/A
Ethics oversight	All of the experimental procedures in the animals were performed in accordance with the NIH Guide for the Care and Use of Laboratory Animals and all protocols were approved by the Thomas Jefferson University IACUC (Protocol 1892). Experiments were carried out in accordance with The Code of Ethics of the World Medical Association (Declaration of Helsinki) for animal experiments.

Note that full information on the approval of the study protocol must also be provided in the manuscript.

Flow Cytometry

Plots

Confirm that:

☒ The axis labels state the marker and fluorochrome used (e.g. CD4-FITC).

☒ The axis scales are clearly visible. Include numbers along axes only for bottom left plot of group (a 'group' is an analysis of identical markers).

☒ All plots are contour plots with outliers or pseudocolor plots.

☒ A numerical value for number of cells or percentage (with statistics) is provided.

Methodology

Sample preparation	details in methods section
Instrument	details in methods section. Data were acquired using a FACSCalibur flow cytometer (BD Biosciences, San Diego CA) and analyzed with CellQuest Pro (BD Biosciences, San Jose, CA) software. T
Software	Data were acquired using a FACSCalibur flow cytometer (BD Biosciences, San Diego CA) and analyzed with CellQuest Pro (BD Biosciences, San Jose, CA) software. T
Cell population abundance	The viability of sorted cell populations was always at least 95%. After sorting, cells were washed with sterile PBS, centrifuged and stored at −80C until assayed.
Gating strategy	After eliminating debris/dead cells (DAPI-positive) and doublet cells by forward- and side-scatter, cells were gated based on CD4 (PerCP-Cy5.5), CD8 (PE-Cy7), CD19 (APC-Cy7), CD11b (Alexa700) and/or GFP fluorescent intensity. A small portion of the cells were incubated without antibodies to gate cells according to their light scattering characteristics, and another small portion of the cells were stained with isotype-matched control reagents to set the maximum threshold for non-specific binding. PBMCs were isolated from double mutant transplanted chimeras (WT(GFP) -> Rag1/R1441G) and sorted for GFP+/CD4+, GFP+/CD8+, GFP+/CD19+ as well as for GFP-negative signal using a FACSAria cell sorter.

☒ Tick this box to confirm that a figure exemplifying the gating strategy is provided in the Supplementary Information.
